# Stimulation and quantification of *Babesia divergens* gametocytogenesis

**DOI:** 10.1186/s13071-016-1731-y

**Published:** 2016-08-08

**Authors:** Marie Jalovecka, Claire Bonsergent, Ondrej Hajdusek, Petr Kopacek, Laurence Malandrin

**Affiliations:** 1 INRA, UMR1300 Biology, Epidemiology and Risk Analysis in Animal Health, CS 40706, F-44307 Nantes, France; 2 LUNAM University, Nantes-Atlantic College of Veterinary Medicine and Food Sciences and Engineering, UMR BioEpAR, F-44307 Nantes, France; 3 Institute of Parasitology, Biology Centre of the Czech Academy of Sciences, CZ-370 05 Ceske Budejovice, Czech Republic; 4 Faculty of Science, University of South Bohemia, CZ-370 05 Ceske Budejovice, Czech Republic

**Keywords:** *Babesia divergens*, Gametocytes, *bdccp* genes, qRT-PCR, Transmission

## Abstract

**Background:**

*Babesia divergens* is the most common blood parasite in Europe causing babesiosis, a tick-borne malaria-like disease. Despite an increasing focus on *B. divergens*, especially regarding veterinary and human medicine, the sexual development of *Babesia* is poorly understood. Development of *Babesia* sexual stages in the host blood (gametocytes) plays a decisive role in parasite acquisition by the tick vector. However, the exact mechanism of gametocytogenesis is still unexplained.

**Methods:**

*Babesia divergens* gametocytes are characterized by expression of *bdccp1*, *bdccp2* and *bdccp3* genes. Using previously described sequences of *bdccp1*, *bdccp2* and *bdccp3*, we have established a quantitative real-time PCR (qRT-PCR) assay for detection and assessment of the efficiency of *B. divergens* gametocytes production in bovine blood. We analysed fluctuations in expression of *bdccp* genes during cultivation in vitro, as well as in cultures treated with different drugs and stimuli.

**Results:**

We demonstrated that all *B. divergens* clonal lines tested, originally derived from naturally infected cows, exhibited sexual stages. Furthermore, sexual commitment was stimulated during continuous growth of the cultures, by addition of specific stress-inducing drugs or by alternating cultivation conditions. Expression of *bdccp* genes was greatly reduced or even lost after long-term cultivation, suggesting possible problems in the artificial infections of ticks in feeding assays in vitro.

**Conclusions:**

Our research provides insight into sexual development of *B. divergens* and may facilitate the development of transmission models in vitro, enabling a more detailed understanding of *Babesia*-tick interactions.

**Electronic supplementary material:**

The online version of this article (doi:10.1186/s13071-016-1731-y) contains supplementary material, which is available to authorized users.

## Background

*Babesia* are protozoan intracellular parasites infecting various vertebrates including humans. All representatives of the genus are cosmopolitan, tick-transmitted pathogens that belong to the most common blood parasites of mammals [1]. *Babesia* forms a sister clade to *Theileria* and together they form a group referred to as Piroplasmida [1, 2]. Babesiosis caused by *Babesia divergens*, the most common blood parasite in Europe, is a disease in human and veterinary medicine that is occurring with increasing incidence [3]. *Babesia* is evolutionarily related to *Plasmodium* [2], the agent of malaria, and both protists share many features in parasite development, such as asexual multiplication in the red blood cells (RBCs) of the vertebrate host and sexual development in the internal organs of the arthropod vector [4, 5].

Gametocytes represent essential developmental sexual stages of apicomplexan life-cycles and, in the case of *Babesia*, they determine the ability to infect the tick [6, 7]. The commitment from asexual growth to sexual maturation already occurs in the blood stream of the vertebrate host [7, 8]. Unlike *Plasmodium*, *Babesia* gametocytes are barely distinguishable from other asexual stages. For this reason, only laborious electron microscopy has reliably described gametocytogenesis in cultures of *Babesia bigemina* [9] or in the blood of hamsters infected with *Babesia microti* [10]. The only case of gametocyte detection by light microscopy was described after stimulation of *B. bigemina* in vitro by addition of xanthurenic acid (XA) [11] or a gut homogenate from fully engorged *Rhipicephalus* (*Boophilus*) *microplus* ticks [12].

*Babesia* gametocytes are also poorly characterized at the molecular level. Several genes, such as heat shock protein 20 and rhoptry-associated protein 1a were believed to be transcribed in *B. bigemina* sexual stages. However, transcription of these genes was later found not to be exclusive for gametocytes and was also detected in other parasite stages [13]. To date, the only molecular assay enabling specific recognition of *Babesia* sexual stages is based on the analysis of a highly conserved family of proteins named CCp [14]. CCp proteins are, in general, characterized by the presence of at least one Limulus coagulation factor C (LCCL) domain [15, 16] and are often involved in cell adhesion [16]. Gene orthologs from the highly-conserved CCp family have been identified in numerous apicomplexan parasites [16–18], including *Babesia* and *Theileria* species [14, 19]. Transcription of *ccp* genes was found to be restricted to gametocytes in vertebrate blood, while translation occurs in the arthropod vector to mediate gamete fertilization [14, 16, 20–24]. Based on post-genomic bioinformatic analyses of *Babesia* and *Plasmodium* genomes, three *bdccp* genes (*bdccp1*, *bdccp2* and *bdccp3*) were thoroughly characterized and described as markers of *B. divergens* sexual stages [14]. The transcripts of *bdccp1*, *bdccp2* and *bdccp3* genes were also detected in gametocytes appearing in cultures of *B. divergens*, *B. bigemina*, *Babesia bovis* and *Theileria equi* [14, 19]. Moreover, antibody targeted to BdCCp2 protein enabled visualization of *B. divergens* sexual stages exclusively in the midgut of *Ixodes ricinus* [22].

Here, we have established qRT-PCR conditions for the assessment of the efficiency of *B. divergens* gametocytes production in cultures in vitro by measuring the expression of *bdccp* genes. This technique is a unique tool to monitor the kinetics of *B. divergens* sexual stages. We analysed changes in expression of *bdccp* genes following variations in cultivation conditions and identified stimuli that significantly increased gametocytemia. Practical applications of our results have the potential to facilitate further detailed research in the field of *Babesia*-tick interactions.

## Methods

### *Babesia divergens*

Strains of *B. divergens* were isolated from bovine blood during the acute phases of babesioses as described earlier [25]. 11 isolates of *B. divergens* from different geographical locations within France were cultivated and cloned by limited dilution [26]. The first two digits in the description of each clone (Additional file 1: Table S1) refer to the French county of origin. Isolate Rouen 87 originated from human blood [27]. *Babesia divergens* isolates were cultivated in vitro in a suspension of bovine erythrocytes obtained from a parasite-free cow (serologically negative and culture tested) as described [25, 26]. Parasitemia was monitored using the commercial Diff-Quik Stain Set (Siemens) and RBC smears.

### Selection of target and reference genes, primer design, DNA extraction and PCR

Previously described *B. divergens* gametocyte-specific sequences of *bdccp1*, *bdccp2*, and *bdccp3* (GenBank Accession Nos. FJ943575.1, FJ943576.1, and FJ943577.1, respectively; [14]) were selected as target genes to quantify the presence of parasite sexual stages (gametocytes) in cultures under various conditions (Table 2). Four reference genes were selected: β-tubulin (*b*-*tubulin*), glyceraldehyde 3-phosphate dehydrogenase (*gapdh*), actin (*actin*) and the small eukaryotic 18S rRNA (*18S*). Sequences were obtained from the *B. divergens* genome database [28] using the nucleotide basic local alignment search tool (BLAST) [29]. All primers were designed using Geneious Pro Trial 5.6.6 software; the sequences and amplicon lengths are summarized in Table 1. The qRT-PCR primers were designed after analysis for polymorphism (see below) particularly towards the conserved regions, especially towards the 3′ end. Negative complementarity of all designed primers with bovine DNA was evaluated *in silico* using BLAST on-line software (blast.ncbi.nlm.nih.gov) and experimentally verified using PCR in a sample containing parasite-free bovine DNA. Genomic DNA (gDNA) was extracted according to the instructions of the Wizard® Genomic DNA Purification Kit (Promega) from frozen infected RBCs as described [30]. PCR was performed using a GoTaq® Flexi DNA Polymerase kit (Promega) with an annealing temperature of 60 °C, using sequencing or qRT-PCR primers according to the manufacturer’s instructions.

**Table 1:** List of oligonucleotides

Gene name and sequence reference	Sequencing primers		qRT-PCR primers	
	Sequence 5′-3′ and amplicon length (bp)		Sequence 5′-3′ and amplicon length (bp)	
*gapdh* (LK934710)	F: TTGACTGTCGATGGTGCTTC	391	F: TACTTACGAGCAGATCGTTGC	140
	R: ACCATGACACAAGCTTCACG		R: CGGCCTTGACATCGAAAATG	
*actin* (LK934710)	F: GCTTTGTTACATTGCCCTCG	437	F: GTCAGCGTATGACGAAGGAG	131
	R: CCTCCTTGGTGATCCACATC		R: CTGGAAGGTGGAAAGGGATG	
*b*-*tubulin* (LK934711)	F: TTCCCCAGACTGCACTTCTT	400	F: GAGTGGATCCCACACAACAC	138
	R: TGTGTACCAGTGAAGGAAGG		R: CATTGCTGTGAATTGCTCCG	
*18S* (FJ944825)	–	–	F: ATGCCTAGTATGCGCAAGTC	131
	–	–	R: AAGCCGACGAATCGGAAAG	
*bdccp1* (FJ943575)	F: GATCGTTCCTCGCTAGCCTAT	639	F: CGCATGCCAGAAAAACAACC	132
	R: TGCACTGATTTACGCAGCTC		R: GCGTCTTTCAGACATCCTCG	
*bdccp2* (FJ943576)	F: GCGGGAGAACATGTAGGATG	701	F: CTGTGAGGCCAACTACTGTG	135
	R: TTCGCAACACAGCTCACAAT		R: AAGTGGTCCACGGTTTTCTG	
*bdccp3* (FJ943577)	F: CCCACCTCCTTTGACTTCAG R: GTGCATCTTGAGCACGAAAA	780	F: GTTGTGGTAAAAGCTGCATGG R: AGAATCGTGACAACTGCCTC	139

### Polymorphism analysis

Polymorphisms in the selected reference and target genes were evaluated in 11 *B. divergens* clonal lines from different locations in France (listed in Additional file 1: Table S1) and compared to the *B. divergens* genome and other available sequences of *bdccp* genes. Partial gene sequences and amplicons for all target and reference genes were amplified with the sequencing primers, purified with ExoSAP-IT® (USB) and sequenced. Sequences were analysed by BioEdit v7.2.5 software.

### Quantitative analysis of expression of *bdccp* genes

Total RNA was extracted by a combination of TRIzol® Reagent (Ambion) and NucleoSpin® RNA extraction kit (Macherey-Nagel). Briefly, 50 μl of pelleted RBCs were mixed with 200 μl of TRIzol and supplemented with 40 μl of chloroform (Sigma-Aldrich), thoroughly vortexed and centrifuged (12,000× *g*, 15 min, 4 °C). The aqueous phase (about 100 μl) was mixed with the same volume of 70 % ethanol and loaded onto the NucleoSpin® RNA extraction kit column. Subsequent procedures were carried out according to the manufacturer’s instructions. Evaluation of quantity and quality of RNA was performed using NanoDrop (Thermo scientific) and Experion (Bio-Rad) analyses. Residual gDNA was removed by DNase digestion with TURBO DNA-free™ Kit (Ambion) according to the manufacturer’s protocol. The absence of residual DNA was verified by lack of amplicon by PCR using qRT-PCR primers for *gapdh*.

Reverse transcription was performed by the SuperScript™III First-Strand Synthesis System for RT-PCR (Invitrogen) using a combination of Oligo(dT) and random hexamers according to the manufacturer’s instructions. qRT-PCR assay was performed using HOT FIREPol® EvaGreen® qPCR Mix Plus (Rox) (Solis BioDyne) in the 7300 Real-Time PCR System (Applied Biosystems). For each biological sample, three technical replicates were performed. Each assay included a standard curve generated from triplicate reactions of a 10-fold serial dilution of template. Based on standard curves, reaction efficiency and specificity were verified for each assay and each gene separately; the value of R2 > 0.98 (the correlating coefficient obtained for the standard curve) and slopes between -3.58 (reaction efficiency 90 %) and -3.10 (110 %) were accepted [31]. For all genes, the dissociation curve analysis was performed to exclude the formation of primer-dimers and to confirm the specificity of primers. The stability of reference gene expression was tested and evaluated by comparisons of all reference genes.

The qRT-PCR results were analysed using Applied Biosystems 7300 Real-Time PCR instrument software. For analysis of the results, a comparative C_t_ (cycle threshold) (2^-ΔΔCt^) method was used [32, 33]. Mean values from technical replicates were assessed and only standard deviation (SD) values ≤ 0.5 were accepted. Target gene expression was normalized using *gapdh* and *actin* and compared using the Student t-test with Welsh’s corrections.

### Analysis of expression of *bdccp* genes in cultures in vitro

All experiments were designed according to the previously published data for *Babesia* and *Plasmodium* [8, 11, 34–37] and carried out in vitro. All experiments were first assayed as pilot experiments using only single replicates of two *B. divergens* clones (2210A G2 and Rouen G11). Based on the results, experiments indicating fluctuations in *bdccp* transcripts were carried out in biological triplicates using *B. divergens* 2210A G2. Detailed descriptions of all experiments are summarized in Table 2. In addition, expression of *bdccp* genes was analysed in 10 bovine clonal lines from different geographical locations (listed in Additional file 1: Table S1); the quantitative analysis was performed 3 days post (culture) initiation (DPI).

**Table 2:** Overview of experimental conditions and resulting effects on expression of *bdccp* genes

Experiment description	*B. divergens* clones	Experiment design	Expression of *bdccp* genes
Continuous culture growth	2210A G2 1802A G8 Rouen G11	The initial parasitemia was set up at 0.1 % and expression of *bdccp* genes was analyzed daily for all five days post culture initiation (DPI); cultivation was performed without medium replacement.	increased*
Long-term cultivation	2210A G2 6903C E2 Rouen F5	*B. divergens* clones 2210A G2 and 6903C E2, were continuously propagated in vitro for ≈ 1 year. Samples before and after long-term cultivation were analyzed; parasitemia was equal for all analysed samples to minimize variations in the expression of *bdccp* genes. Expression of *bdccp* genes by *B. divergens* clone Rouen F5 was analyzed by PCR using gDNA and cDNA.	decreased
Imidocarbe treatment	2210A G2 Rouen G11	The range of efficient doses of both drugs was determined following parasite growth monitoring in vitro for 48 h [66] to select effective concentrations of drugs (imidocarbe 179.5 nM, 359 nM and 718 nM; atovaquone 10 nM, 40 nM and 75 nM). The culture without drug treatment was used as a control. The effect of drug treatment was measured 2 DPI; starting parasitemia was 2 %.	increased*
Atovaquone treatment			increased or decreased* (concentration dependent)
Altered cultivation temperature and air environment	2210A G2	XA was added at 100 μM concentration and its effect was tested after 24 h of parasites cultivation either under standard (37 °C, 5 % CO2) or altered conditions (28 °C, air). As a control, cultures without XA were used. A starting parasitemia was set up 6 % in order to reach > 10 % parasitemia level (experiment design setting taken from [11]).	increased*
XA addition			increased*
Combination of altered cultivation and XA addition			increased*
Co-infection	2210A G2 Rouen G11 7101A D11	Different clonal lines were mixed in the same ratio and expression of *bdccp* genes was analysed in cultures cultivated for 24 h and 48 h. As a control, clones were cultivated independently; starting parasitemia was 2 %.	not affected
RBCs lysate addition	2210A G2 Rouen G11	Lysate of uninfected RBCs was added into the culture to simulate cultivation medium corresponding with 10 % parasitemia. Analyses were performed after 24 and 48 h of cultivation; the control was represented by a culture without lysate addition; starting parasitemia was 2 %.	not affected
Hematocrit increase	2210A G2 Rouen G11	Hematocrit increase was simulated by doubling the quantity of RBCs in the medium and analyses were performed after 24 h and 48 h of cultivation; standard in vitro culture was used as a control; starting parasitemia was 2 %.	not affected
High parasitemia maintenance	2210A G2 Rouen G11	Analyses were performed at the starting point (0 DPI), where parasitemia was starting at 10 %, and 1 and 2 DPI. Media were changed daily.	not affected
Cultivation without FCS	2210A G2 Rouen G11	Altered cultivation conditions (cultivation in medium without FCS) were maintained for 24 h in culture with 10 % parasitemia. Analyses were performed 0 and 1 DPI; starting parasitemia was 2 %.	not affected

*Abbreviations*: *XA* xanthurenic acid, *RBCs* red blood cells, *FCS* fetal calf serum, *DPI* days post initiation

*P  < 0.05

### Statistical analysis

Statistical analyses were performed in R (version 3.2.2), a software environment for statistical computing (https://www.r-project.org/), using the Student t-test with Welsh’s correction or ANOVA followed by Tukey’s multiple comparisons test, assuming that the Bartlett test of homogeneity of variances was passed. Graphs were designed in GraphPad Prism (version 6). For graphical representations of the results and statistical analyses, mean values (± standard deviation, SD) from three biological replicates (independent experiments) were assessed.

## Results

### Analysis of gene polymorphisms

Previously, polymorphisms had not been detected in *18S* rDNA sequences from several *B. divergens* isolates [30]. Hence sequence FJ944825 (GenBank) was used as an *18S* rDNA reference gene. For other reference genes, we did not detect any polymorphisms in the *b*-*tubulin* gene and only two synonymous substitutions in *gapdh* and *actin* genes. *bdccp1* was found to be highly conserved (one synonymous substitution) compared to *bdccp2* (6 substitutions in the coding regions, 2 non-synonymous, resulting in 5 different sequences) and *bdccp3* (7 substitutions, 2 localized in introns, all synonymous, resulting in 7 different nucleotide sequences) genes (Additional file 2: Figure S1). The qRT-PCR primers were designed only in the conserved regions.

### Optimization of expression of *bdccp* genes

qRT-PCR was optimized as recommended by MIQE [31] for reference (*18S*, *gapdh*, *actin*, *b*-*tubulin* and *18S*), as well as for target (*bdccp1*, *bdccp2*, *bdccp3*) genes. Standard curves of reference, target genes and qRT-PCR parameters are summarized in Additional file 3: Figure S2. Comparisons between reference genes using C_t_ values showed that *gapdh* and *actin* were the most stably expressed (Additional file 4: Figure S3) and these genes were therefore selected as references for further analyses. Sample normalization to *gapdh* or *actin* were consistent and no significant differences were recorded.

### Expression of *bdccp* genes under standard cultivation conditions

**Fig. 1:**
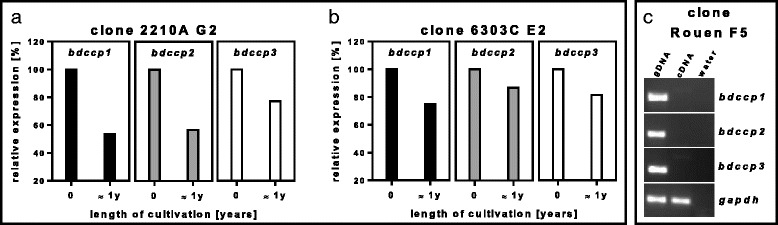
Long-term cultivation. Influence of long-term [≈1 year (1y)] cultivation on the relative expression of *bdccp* genes during continuous growths of *B. divergens* clones 2210A G2 and 6903C E2 (**a**, **b**). Gene expression was normalized using the *gapdh* reference gene. The expression in samples collected at the beginning of long-term cultivation (0) was set at 100 %. **c** Loss of expression of *bdccp* genes after several years of cultivation of *B. divergens* clone Rouen F5 tested by PCR

All field bovine clonal lines uniformly expressed *bdccp* genes, with the *bdccp*1 gene having the lowest level of transcripts and the *bdccp3* gene having the highest level (Additional file 5: Figure S4). The influence of long-term cultivation on expression of *bdccp* genes was measured for three clonal lines. The decrease in transcription of *bdccp* genes was also noted in the long-term (≈1 year) cultures of 2210A G2 and 6903C E2 clones (Fig. 1a, b). *B. divergens* clone Rouen F5, propagated in vitro for several years, had already lost the ability to express *bdccp* genes (Fig. 1c). The presence of gametocytes in the original sample of *B. divergens* clone Rouen F5 was confirmed by PCR (data not shown). Asexual multiplication of the parasite was not affected, as demonstrated by the continuous presence of parasitemia in blood smears as well as by expression of the *gapdh* reference gene.

**Fig. 2:**
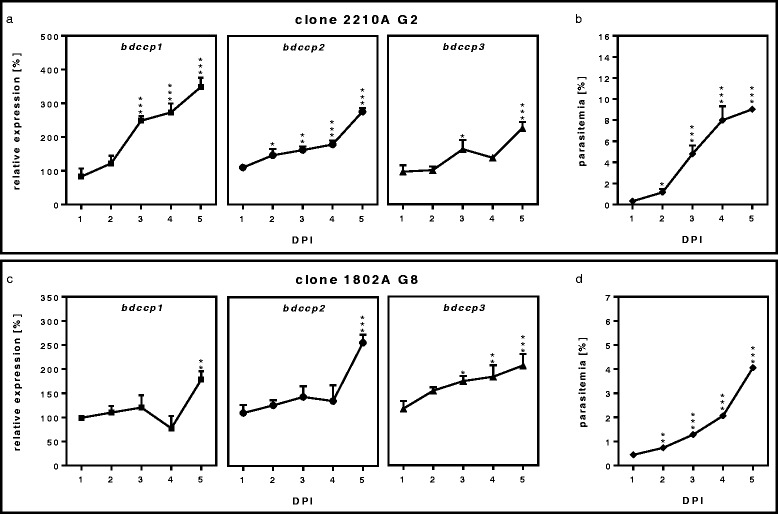
Continuous culture growth. Relative expression of *bdccp* genes (**a**, **c**) and parasitemia levels (**b**, **d**) during the continuous growth of *B. divergens* clone 2210A G2 and 1802A G8. Gene expression was normalized using the *gapdh* reference gene. The results represent means of three independent biological replicates, where the highest expression in the individual replicate 1 DPI was set at 100 % and all other values were expressed relative to this. *P < 0.05; **P < 0.01; ***P < 0.001 (compared to 1 DPI). Error bars indicate SD

An increase in expression of *bdccp* genes was recorded during continuous growth of all strains of *B. divergens*. Using clone 2210A G2, increased transcription of *bdccp1*, *bdccp2* and *bdcpp3* genes was observed in a pilot experiment (increase 4.7, 4.1 and 3.3 times, respectively; Additional file 6: Figure S5) and confirmed by repeated analysis in biological triplicates, where *bdccp1* and *bdccp2* levels significantly increased from 3 DPI (*F*_(4, 10)_ = 66.02, *P* < 0.001) and 2 DPI (*F*_(4, 10)_ = 73.85, *P* = 0.033), respectively. After 5 days of cultivation, *bdccp1* and *bdccp2* gene expression increased 3.5 (*F*_(4, 10)_ = 66.02, *P* < 0.001) and 2.7 (*F*_(4, 10)_ = 73.85, *P* < 0.001) times, respectively. The level of the *bdccp3* transcript significantly increased only 3 DPI (*F*_(4, 10)_ = 26.26, *P* = 0.012) and 5 DPI (2.3 times, *F*_(4, 10)_ = 26.26, *P* < 0.001) (Fig. 2a). A similar pattern was observed for *B. divergens* clone 1802A G8, where expression of *bdccp1* and *bdccp2* genes increased significantly 5 DPI: 1.8 (*F*_(4, 10)_ = 11.79, *P* = 0.003) and 2.6 (*F*_(4, 10)_ = 22.81, *P* < 0.001) times, respectively. A significant increase in expression of the *bdccp3* gene was recorded from 3 DPI (*F*_(4, 10)_ = 11.11, *P* = 0.017) and increased 2.1 times on 5 DPI (*F*_(4, 10)_ = 11.11, *P* < 0.001) (Fig. 2c).

### Expression of *bdccp* genes under stress conditions

**Fig. 3:**
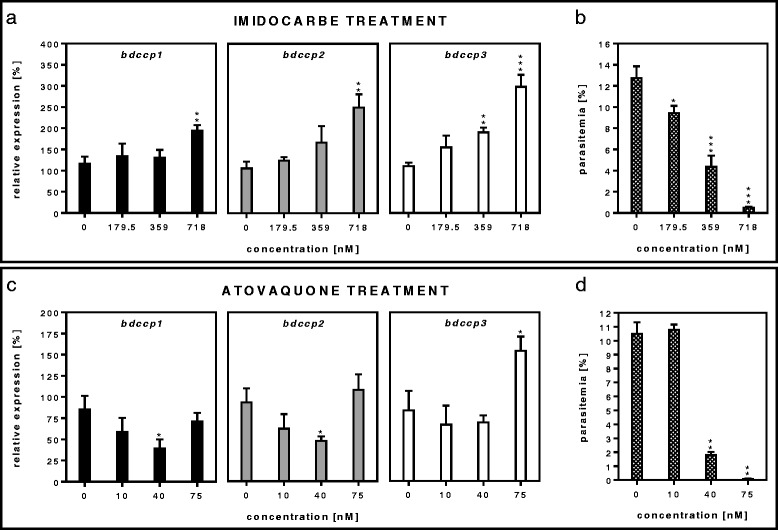
Imidocarbe and atovaquone treatment. The effect of imidocarbe (**a**) and atovaquone (**c**) treatment on the relative expression of *bdccp* genes and corresponding parasitemia (**b**, **d**) of *B. divergens* clone 2210A G2. Gene expression was normalized using the *gapdh* reference gene. The results represent means of three independent biological replicates, where the highest expression in the individual replicate of untreated culture (0) was set at 100 % and all other values were expressed relative to this. *P < 0.05; **P < 0.01; ***P < 0.001 (compared to the untreated culture). Error bars indicate SD

**Fig. 4:**
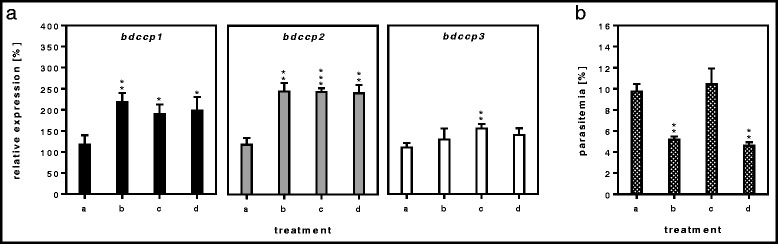
XA treatment and altered cultivation conditions. The effect of XA (xanthurenic acid) treatment and altered cultivation conditions on the relative expression of *bdccp* genes (**a**) and corresponding parasitemia levels (**b**) of *B. divergens* clone 2210A G2 culture. (*a*): untreated culture cultivated at 37 °C, 5 % CO_2_; (*b*): untreated culture cultivated at 28 °C, air; (*c*): XA treated culture cultivated at 37 °C, 5 % CO_2_; (*d*): XA treated culture cultivated at 28 °C, air. Gene expression was normalized using the *gapdh* reference gene. The results represent means of three independent biological replicates, where the highest expression in the individual replicate of untreated culture (*a*) was set at 100 % and all other values were expressed relative to this. *P < 0.05; **P < 0.01; ***P < 0.001 (compared to the untreated culture). Error bars indicate SD

Simulation of stress conditions in *B. divergens* in vitro by drug treatment resulted in a significant increase in expression of *bdccp* genes (Figs. 3 and 4). Imidocarbe, a drug routinely used in veterinary medicine to treat babesiosis [3], almost completely inhibited parasite growth at a concentration of 718 nM (*t*_(4)_ = 17.31, *P* < 0.001). At this concentration, expression of all *bdccp1*, *bdccp2* and *bdcpp3* genes were significantly increased: 1.8 (*t*_(4)_ = -6.36, *P* = 0.004), 2.5 (*t*_(4)_ = -6.96, *P* = 0.007) and 3.0 (*t*_(4)_ = -11.07, *P* < 0.001) times, respectively, but simultaneously, overall parasitemia was reduced more than 10 times, compared to the control. Treatment with 359 nM imidocarbe showed a moderate killing effect (*t*_(4)_ = 9.19, *P* < 0.001), but resulted in a significant increase (1.9 times, *t*_(4)_ = -9.78, *P* = 0.005) in expression of only *bdccp3*. 179.5 nM imidocarbe decreased parasitemia by only 1.3 times (*t*_(4)_ = 4.40, *P* = 0.018) with no effect on expression of *bddcp* genes (Fig. 3a, b).

Atovaquone, another effective anti-babesial drug that induces cellular oxidative stress and is commonly used in malaria and human babesiosis treatments [38], caused a significant reduction in growth of *B. divergens* at concentrations of 40 nM (moderate inhibitory effect, *t*_(4)_ = 17.88, *P* = 0.002) and 75 nM (complete inhibitory effect, *t*_(4)_ = 22.02, *P* = 0.002). At a concentration of 40 nM, drug treatment resulted in significantly reduced expression of *bdccp1* and *bdccp2* genes: 2.2 (*t*_(4)_ = 4.19, *P* = 0.017) and 2.0 times (*t*_(4)_ = 4.55, *P* = 0.032), respectively, whereas at 75 nM, atovaquone significantly increased *bdccp3* transcript levels (1.8 times, *t*_(4)_ = −4.28, *P* = 0.016) (Fig. 3c, d).

A reduction in cultivation temperature from 37 to 28 °C in combination with a change in environmental conditions from 5 % CO_2_ to an air atmosphere, resulted in significant inhibition (*t*_(4)_ = 9.84, *P* = 0.004) of parasite division as well as in a significant increase in expression of *bdccp1* (*t*_(4)_ = -5.57, *P* = 0.005) and *bdccp2* (*t*_(4)_ = -8.32, *P* = 0.002) genes (Fig. 4a, b). Treatment with XA, a metabolic intermediate of tryptophan degradation, has been proposed to increase the development of sexual stages in *B. bigemina* in vitro [11]. In our experiments with XA treatment and cultivation at 37 °C and 5 % CO_2_ we identified conditions that significantly increased expression of all *bdccp1*, *bdccp2* and *bdcpp3* genes: 1.9 (*t*_(4)_ = -3.97, *P* = 0.017), 2.4 (*t*_(4)_ = -11.97, *P* < 0.001) and 1.6 (*t*_(4)_ = -5.27, *P* = 0.006) times, respectively (Fig. 4a) without any inhibitory effect on culture growth (Fig. 4b). Combining XA treatment with altered cultivation conditions (28 °C, air atmosphere) resulted in significantly increased expression of *bdccp1* (2.0 times, *t*_(4)_ = -3.54, *P* = 0.029) and *bdccp2* (2.4 times, *t*_(4)_ = -8.39, *P* = 0.001) genes but culture growth was significantly inhibited (*t*_(4)_ = 10.80, *P* = 0.002) (Fig. 4b). All other stress factors tested did not result in a significant increase in expression of *bdccp* genes (Table 2).

## Discussion

The production of gametocytes in the host blood is a prerequisite for successful parasite transmission to the arthropod vector. *Plasmodium* gametocytemia, which could be quantified by simple light microscopy [39, 40], was demonstrated to closely correlate with mosquito infection [41–44]. However, such a simple morphological identification is not possible for *Babesia* gametocytes, preventing controllable infections of ticks. Based on similarities between these two parasites [2], we presumed that similarly to *Plasmodium*, changes in the expression of *Babesia* sexual stage-specific *bdccp* genes would correlate with actual numbers of gametocytes in the total intra-erythrocytic parasite population. Using previously described sequences of *bdccp1*, *bdccp2* and *bdccp3* genes [14], we have developed a qRT-PCR assay to detect and quantify gametocyte densities in *B. divergens* cultures in vitro. Based on comparisons between the reference genes we chose *actin* and *gapdh* as references for our assays (Additional file 4: Figure S3). The *18S* rDNA exhibited lower stability than *actin*, *gapdh* or *b*-*tubulin*. This result differs considerably from the generally accepted view that *18S* rDNA is one of the most stably expressed genes [45, 46].

The selection of specific target and reference gene primers, universal for most of the *B. divergens* strains, was absolutely critical for further reliable assessment of the gametocytes production efficiency by qRT-PCR. Despite the fact that CCp proteins are presumed to be conserved among the apicomplexan parasites [16, 19, 20], no data were available about single nucleotide polymorphisms of *ccp* genes among various strains within one species. We demonstrated that between the 11 *B. divergens* clonal lines, nucleotide sequences of *ccp* genes varied, especially for *bdccp2* and *bdccp3* genes (Additional file 2: Figure S1). On the contrary, the *bdccp1* gene was highly conserved. The sequences of reference genes seemed to be highly conserved. Some studies questioned the suitability of *actin* and *gapdh* reference genes because of their variabilities [45], but our results did not support this (Additional file 2: Figure S1) and confirmed their suitability.

The appearance of gametocytes in the blood is a crucial event that it is still not fully understood. Referring to the recent knowledge on *Plasmodium*, commitment towards the sexual development occurs randomly, asynchronously and is governed by the genetic and environmental factors [47], as demonstrated by detailed studies performed on *Plasmodium* (see reviews [8, 36, 37, 48–50]). To date, only one study has been dedicated to this subject in *Babesia* (*B. bigemina*) [11].

We tested the effects of various factors and conditions on gametocytogenesis in *B. divergens* cultures. Our results demonstrated the ability of *B. divergens* to produce gametocytes (measured by expression of *bdccp* genes in several bovine strains; Additional file 5: Figure S4) after a short term cultivation. On the contrary, long-term cultivation led to a significant decrease or even absence of expression of *bdccp* genes (Fig. 1), suggesting that these cultures had halted production of gametocytes and were probably no longer infectious for ticks. Similarly to *Babesia*, the disappearance of gametocytes from long-term maintained *Plasmodium falciparum* cultures has also been described (reviewed in [37]), therefore only fresh cultures with low passage numbers should be used for tick or mosquito infection studies.

The enhancement of *Babesia* sexual commitment was observed after several days of cultivation without medium changes, but minor variations were recorded in the *bdccp* genes expression of various *B. divergens* strains (Fig. 2, Additional file 6: Figure S5). Such phenomena could be explained by the stochastic differentiation mechanism, that was previously reported for *Theileria* [51]. A rapid expansion of a *Plasmodium* population (intensive multiplication of asexual stages) also resulted in an increase in gametocytogenesis [34, 35]. A possible explanation of this phenomenon is the accumulation of metabolites under stress conditions, as high parasitemia or regular medium exchanges did not alter levels of *bdccp* transcripts (Table 2). This change is probably induced by the accumulation of metabolic waste in the blood, as an addition of a lysis solution of healthy RBCs had no significant effect (Table 2). Nevertheless, hemolysis products of both infected and healthy RBCs influenced production of gametocytes of *P. falciparum* in vitro as well as *Plasmodium chabaudi* in vivo [52, 53]. As previously shown, mixed population of *Plasmodium* species could result in an increase of gametocytemia and promoted more successful transmission into the vector [36, 54, 55] despite some contradictory results [56]. We did not observe this phenomenon for *B. divergens* isolates (Table 2), however the choice of strains could greatly influence results, depending on their modes of interaction (neutral or synergistic instead of antagonistic).

Addition of inhibitory drugs certainly represents a stressful condition for the parasite. Numerous experiments performed on *Plasmodium* proved that treatment with anti-malarial drugs had an effect on the recruitment of gametocytes, both in vivo and in vitro (reviewed in [8, 48, 50, 57]). We have tested the effects of imidocarbe and atovaquone, the widely used anti-babesial drugs. Imidocarbe has been used for over 20 years as the drug of choice for the treatment and prophylaxis of animal babesiosis [58]. The mode of action of imidocarbe still remains unclear, although disruption of polyamine metabolism or a blockage of inositol influx into parasitized cells was proposed [58]. In our experiment, imidocarbe treatment significantly increased all three *bdccp* transcripts, while overall parasitemia was greatly decreased (Fig. 3). This implies that this drug either stimulated sexual commitment to the sexual pathway or has a lower impact on gametocytes as they are metabolically less active compared to asexual stages.

Atovaquone is widely used to treat babesiosis (and malaria) in humans [38] and causes oxidative stress in the parasite by inhibition of the mitochondrial electron transfer [59]. This drug displayed remarkable activity against asexual stages. In gametocytes, only *bdccp3* gene transcription was significantly increased (Fig. 3). It was previously demonstrated that atovaquone treatment had different effects on the various maturation stages of *P. falciparum* gametocytes [60, 61]. Therefore, we speculate that differences in gene expression of *bdccp1* and *bdccp2* compared to *bdccp3* upon atovaquone application could be also related to the age of *Babesia* gametocytes.

Physical or chemical alterations of the parasite environment that mimic transition from the blood stream to the vector gut (temperature decrease from 37 to 28 °C, CO_2_ decrease from 5 % to air environment and addition of a gut homogenate from fully engorged ticks or XA) have been shown to have an effect on the *Babesia* sexual development [11, 12, 62]. We observed a similar stimulation of *B. divergens* sexual commitment after changes in the cultivation environment and/or XA addition using analysis of *bdccp* genes transcription. However, no apparent cumulative effect was observed when combining these stimuli. We demonstrated that XA addition into the culture under standard cultivation conditions (37 °C and 5 % CO_2_) significantly stimulated *B. divergens* sexual commitment without inhibiting parasite growth. This is in contrast to previously published results for *B. bigemina*, where no change in gametocyte development occurred upon XA treatment of a culture propagated under the same conditions [11]. In mosquitoes, XA naturally produced inside the gut is able to induce gamete formation and exflagellation of *Plasmodium* parasites [63, 64]. As exflagellation does not occur in the *Babesia* life cycle, the exact effect of XA on *Babesia* sexual development remains to be elucidated. It is not verified yet whether XA is produced inside the tick gut. If so, one can speculate that *Babesia* gametocytes might be stimulated in the host blood by the tick gut contents regurgitated during the week-long feeding of the adult tick female. This hypothesis could be supported by the studies demonstrating that gametocyte development was stimulated after addition of tick gut homogenate [12, 62]. Further investigation is needed to provide an unequivocal answer.

## Conclusion

Compared to *Plasmodium*, sexual development of *Babesia* is poorly understood. Our research provided insight into sexual development of *B. divergens* during either standard cultivation conditions in vitro or cultivation under stress by different stimuli. Using our newly introduced quantification assay of *bdccp* genes transcripts by qRT-PCR we have shown that levels of gametocytes fluctuate during *B. divergens* culture in vitro and identified conditions that significantly increased the transcription of *bdccp* genes (and thus gametocytemia). By setting these conditions we should be able to perform studies focusing on the transmission and persistence of *Babesia* in the tick vector using an artificial membrane feeding system of ticks [65]. Research aimed to identify and characterize molecular mechanisms of interaction between the parasite and the tick vector could accelerate discovery of effective therapies or vaccines blocking *Babesia* transmission.

## Abbreviations

BLAST, basic local alignment search tool; bp, base pairs; cDNA, complementary DNA; C_t_, cycle threshold; DPI, days post (culture) initiation; FCS, fetal calf serum; *gapdh*, glyceraldehyde 3-phosphate dehydrogenase; gDNA, genomic DNA; LCCL, Limulus coagulation factor C; qRT-PCR, quantitative real-time PCR; RBCs, red blood cells; SD, standard deviation; XA, xanthurenic acid

## Additional files

Additional file 1: Table S1. List of *B. divergen*s strains used in this study (a – gene polymorphism analysis, b – analysis of expression of *bdccp* genes). (DOC 37 kb) Additional file 2: Figure S1. Consensus partial nucleotide sequences of genes used for design of qRT-PCR primers. (A) *gapdh*, (B) *actin* (C), *b*-*tubulin*, (D) *bdccp1*, (E) *bdccp2*, (F) *bdccpp3*. The localization of introns and primers are indicated in yellow and blue, respectively. The sequences were obtained from 11 different biological clones from France: Rouen F5 (human origin), 1406B F10, 1505B F4, 1705A G10, 2705A E11, 3601B E2, 4201B D4, 4903A D11, 5012A G3, 7904B G11, 8706A G8 and from the *B. divergens* genome sequence. As previously reported, sequencing of the *18S* rDNA revealed no variation within *B. divergens* [30], so sequence FJ944825 was taken as reference. Variable nucleotides are highlighted and the corresponding clone(s) and modifications are indicated below each consensus sequence using a color code. (PDF 10 kb) Additional file 3: Figure S2. Optimization of qRT-PCR. Standard curves of reference and target genes (A) and qRT-PCR parameters (B). C_t_ = cycle threshold, R2 = correlation coefficient. (PDF 181 kb) Additional file 4: Figure S3. Comparison of stability of reference genes. Reference genes were evaluated by comparisons of all reference genes using C_t_ values. The first sample in each gene analysis was set at 100 % and all other values were normalized to this. (PDF 66 kb) Additional file 5: Figure S4. Relative expression of *bdccp* genes in various bovine clonal lines of *B. divergens*. Gene expression was normalized using the *gapdh* reference gene. Expression in the clone 2210A G2 was set at 100 % and all other values were expressed relative to this. (PDF 49 kb) Additional file 6: Figure S5. Continuous culture growth. Relative expression of *bdccp* genes (A) and parasitemia levels (B) during the continuous growth of *B. divergens* clone 2210A G2. Gene expression was normalized using the *gapdh* reference gene. The expression in the highest individual replicate 1 DPI was set at 100 % and all other values were expressed relative to this. (PDF 48 kb)
