# Supplementary material for: Stimulation and quantification of Babesia divergens gametocytogenesis
Source: Parasit Vectors. 2016 Aug 8;9:439. doi: 10.1186/s13071-016-1731-y (PMC4977898; doi:10.1186/s13071-016-1731-y)
Supplement: Additional file 1: Table S1. — List of B. divergens strains used in this study (a – gene polymorphism analysis, b – analysis of expression of bdccp genes). (DOC 37 kb) [file 13071_2016_1731_MOESM1_ESM.doc]

**Table S1. List of *B. divergen*s strains used in this study (a – gene polymorphism analysis, b – analysis of expression of *bdccp* genes).**

| **Strain** | **County in France** | **Use** |
| --- | --- | --- |
| Rouen 87 (clones F5 and G11) | Seine Maritime | a-b |
| 1406B F10 | Calvados | a-b |
| 1505B F4 | Cantal | a |
| 1705A G10 | Charente-Maritime | a |
| 1802A G8 | Cher | b |
| 2210A G2 | Côtes d'Armor | b |
| 2305B E7 | Creuse | b |
| 2705A E11 | Eure | a-b |
| 3601B E2 | Indre | a |
| 4201B D4 | Loire | a |
| 4903A D11 | Maine et Loire | a |
| 5005A G5 | Manche | b |
| 5008A D10 | Manche | b |
| 5012A G3 | Manche | a |
| 5608A D10 | Morbihan | b |
| 6903C E2 | Rhône | b |
| 7101A D11 | Saône et Loire | b |
| 7904B G11 | Deux Sèvres | a |
| 8706A E8 | Haute Vienne | a |
