# Supplementary material for: Stimulation and quantification of Babesia divergens gametocytogenesis
Source: Parasit Vectors. 2016 Aug 8;9:439. doi: 10.1186/s13071-016-1731-y (PMC4977898; doi:10.1186/s13071-016-1731-y)
Supplement: Additional file 2: Figure S1. — Consensus partial nucleotide sequences of genes used for design of qRT-PCR primers. (A) gapdh, (B) actin (C), b-tubulin, (D) bdccp1, (E) bdccp2, (F) bdccpp3. The localization of introns and primers are indicated in yellow and blue, respectively. The sequences were obtained from 11 different biological clones from France: Rouen F5 (human origin), 1406B F10, 1505B F4, 1705A G10, 2705A E11, 3601B E2, 4201B D4, 4903A D11, 5012A G3, 7904B G11, 8706A G8 and from the B. divergens genome sequence. As previously reported, sequencing of the 18S rDNA revealed no variation within B. divergens [30], so sequence FJ944825 was taken as reference. Variable nucleotides are highlighted and the corresponding clone(s) and modifications are indicated below each consensus sequence using a color code. (PDF 10 kb) [file 13071_2016_1731_MOESM2_ESM.pdf]

A- GADPH partial gene consensus sequence (nt 666157 to 665789 in LK934710)

GGTGCTTCCCGCGGTGGCAAGGACTGGAGGGCTGGTCGGTGCGCCGGTGCCAACATCATCCCTGCTTCCACTGGTGCCGCCAA
GGCTGTTGGCAAGGTCATTCCTGAGTTGAACGGCAAGCTCACTGGTATGGCTTTCAGGGTACCTACTCCCGATGTCAGTGTTGT
TGACTTGACCTGCAAGCTTGCTAAGCCAGCTACTTACGAGCAGATCGTTGCTGCTGTCAAGGAGGCTGCTGAGGGTGAGCTTA
AGGGTGTGTTGGGCTGGACCGAGGACCAGCTGGTATCCCAGGACTTCGTGCACGACCGCAGGTCTAGCATTTTCGATGTCAAG
GCCGGTATTGCTCTGAACGACACCTTCGTGAAGCTT

A in 1705A G10; G in 4201B D4

B- Actin partial gene consensus sequence (nt 1289265 to 1288852 in LK934710)

TGCCCTCGACTTCGAGGAGGAGATGAACTCTGCCTCCTCATCCAGTGAGATTGAGAAGTCGTACGAGTTGCCTGATGGAAACAT
CATCACTGTTGGCAACGAGCGGTTCAGGTGCCCTGAGGTGTTGTTCCAGCCCACCTTCATTGGTATGGAGTCTCCTGGTATCCA
CACCACAACCTTCAACTCCATTGCCCGCTGTGATCTCGACATCCGCAAGGACCTCTACTCAAACGTCGTGTTGTCTGGAGGTAC
GACGATGTACGAGGGTATCGGTCAGCGTATGACGAAGGAGCTGAATGCCTTGGTTCCCAGCACCATGAAGATCAAGGTTGTTG
CCCCTCCGGAGCGGAAGTACTCTGTCTGGATTGGTGGTTCGATTCTATCATCCCTTTCCACCTTCCAGCAGATGTGGAT

C in 1705A G10 ; C in Rouen F5, 4903A D11, 8706A E8 and 1705A G10

C- Beta Tubulin partial gene consensus sequence (nt 833653 to 833244 in LK934711)

TGCACTTCTTCATGATTGGATTCGCACCACTCACGAGCAGGGGAAGCCAGCAGTACCGTGCCCTCACGGTCGCCGAACTTACA
CAGCAAATGTTCGACGCCAAGAACATGATGTGTGCGTCTGACCCCAGGAGGGGGAGGTATCTCACAGCCTGCGCCATGTTCAG
AGGGCGCATGAGCACCAAGGAAGTCGACGAACAGATGTCGATGGTCCAGAACAAAAACGCCTCATACTTTGTGGAGTGGATCC
CACACAACACAAAGTCAAGCGTATGTGACATCCCACCAAAGGGGCTCAAGATGGCCGTTACATTCGTAGGAAACTCAACCGCC
ATCCAGGACATGTTCAAAAGGGTATCGGAGCAATTCACAGCAATGTTCAGGCGTAAGGCCTTCCTTCACTGGTACACA

D- BdCCp1 partial gene consensus sequence (nt 4504 to 5124 in FJ943575.1)

CTCGCTAGCCTATTTAAGGCTGCTGATTATGAATGCAAGGCAGATATCACTGACGACAAAACGCTGGGATTCAACCTGGGGCTC
AAGTCCAGTGCGTGCGACTCTTGCTACTTTGACACATTCCAGGTATCACCAGACCCAATTCAGACCGTTGTACCAGCGACTAAT
TTCGTCGAAGAGTTCTCCAGTGCCAATGTATGGAAGCCATGCGTGGAAAACGTCCATATATTAAGCAGAGTGGCATTATGCAAG
CAGATGTATCGTAATAAAGGAAACTCGCACGTCTGTGCAGAAAGTTTCTGTACCCCTTGTTGCGCCTATCACACTGTGCTGCTG
GGAGACGGGCATCAGCAAATGTGTGTCGACGCATGCCAGAAAAACAACCACCGTGTGAACGCTTACCTAGAGAATTTCCTGTC
GTACGTCAACAGCTGTGTGTCGCTAAAGGGGGTGGGATACGATCACTGTGATGGGGTAAGTATGGCGCCAAATAGCTTGTTCAT
AATGCTGTAGGATCGAGGATGTCTGAAAGACGCATGCAGACTTTGCTGCACCAGTGGGCACCAGGAGAACAGCCTGCTTGACA
AGCAACTTGTGCTTTTGGAAACGTCGAGCTGCGTA

T in 8706A E8

E- BdCCp2 partial gene consensus sequence (nt 4336 to 5018 in FJ943576.1)

CATGTAGGATGATTCAGATGCACAAGGGTAGCCGCATCGTCCTAGCGGAAACTTCCATGAAGGGAATGGTCATCGGTGCGTGGA
ATACCGTTGAACTGGTCTTCAAACCTGACGCTGTAACTGCTTCTATCGGCACTAATCTACTAGAGGCAGTGTTCATTAACACAGC
CGTGGCAGAGGATGTGCGTGAGCGCGGAACTGTCGGCATTTACAGTCTTGGCTGTGCGGGGTGTGCCTTCGCTGGTATCTCACT
TACCCCCAACTACGCCGTCGGGTATGCCAAGATGCTGACCAGCTCTGAGAACGCCAACACGGGTAGCAGGTCGGCACGGGAG
GAGCAGTGCATGGCAATCGACCGCCTGGACCATTGCAAGGCCATCGCATTCAGCCAACGTGGCAAACTGTGAGGCCAACTACTG
TGCCTTCTGCTGCGAACAGCAGCATAGGGATGCACCAGGGCCTCAGGATGTGTGTTACAACAGATGCAGAAACTTAGATCATGC
GGCCGTGCTACTGCAGAAAACCGTGGACCACTTCTGGCGCAGCTGCGCACCGAATAGCAACAGCGAAACAAATGATCAAAAA
GGTGGGTTCAATGACTTAGATATAATGCGTAACATTACTCTCACAGGATCAGTTGGTGCAATCGAATACGGAACCGATGCTGTGG
GGGATTGTGAGCTG

T in FJ943576.1; G and T in 1406B F10, 1505B F4, 1705A G10, 7904B G11 and FJ943576.1; C and C in 1406B F10, 1505B F4, 1705A G10, 4903A D11, 7904B G11 and FJ943576.1; G in 1705A G10

Non synonymous substitutions : A (Thr) to G (Ala), T (Ser) to C (Pro).

F- BdCCp3 partial gene consensus sequence (nt 3022 to 3780 in FJ943577.1)

TGACTTCAGTTTGCAGGTTTGGTCTGTCTTTTGCGTGGTCTATACTTGTTTAGGGCGATTTAGTCCTTGGGAAGTCTGCGGAGAT
TGATTCTGACTTTTTTATCGGCCAGATCCTGTCGCTGGAGATTTTCGATTACGTCATGTCTCCAGATCAGGTGGAGCACTACAGC
CGTCTGTTACAGGCTTCTAGCGACAATCGGATCAACTCTCGCCACTCTCCAACTCGTATGACTGTTGATGGCAATCTGTGTCTGA
GCAGCTGCGTGAAGATGCGCGCTCCATCGCAAAACGCGGGTTTGCTTGCGGGCCCTACCAATCCTGCTATCCATTTGGGTTGCC
ACGATTCGCTTGAGAACGAGCGTTTCAATGGCGCTACTGGGCATGAGTTTTTGGTGAGTTGTGGTAAAAGCTGCATGGACCCTG
ATTTGGTCCTTAACGGCAGCAAGGTGTACACCCCCGACTCTTCTATATGCAAGGCTGCCATCCACTGTGGTTGCGATTCCCAAGG
AAGGCGGCGAGGCAGTTGTCACGATTCTACATGGTCGCGATAGCTACGACCACTGCCTGGGTCATTACGGTAGGTGCACTTTAG
ATTCTACATTCATACGCTTCAGGGGTGCTGAGCAACAAGTCTAGCATTCCCCATATGCGTTCGTTTTCTGTGCGCAGAGCCCCCA
AGGTGTTGACTCTGTCGTGTCAAGGTAAGCGTGCCGATTTGTGTTTCTGTATGACTGCTTGCAGACACCGCTATTTTCGTGCT

T in 1705A G10, 4201B D4, 5012A G3, and 7904B G11; T and T in 3601B E2, 5012A G3 and 8706A E8 ; G in FJ943577.1 ; A in 3601B E2 and 8706A E8 ; C in 4903A D11 ; T in 4201B D4.
