# Supplementary figures and images for: Stimulation and quantification of Babesia divergens gametocytogenesis
Source: Parasit Vectors. 2016 Aug 8;9:439. doi: 10.1186/s13071-016-1731-y (PMC4977898; doi:10.1186/s13071-016-1731-y)

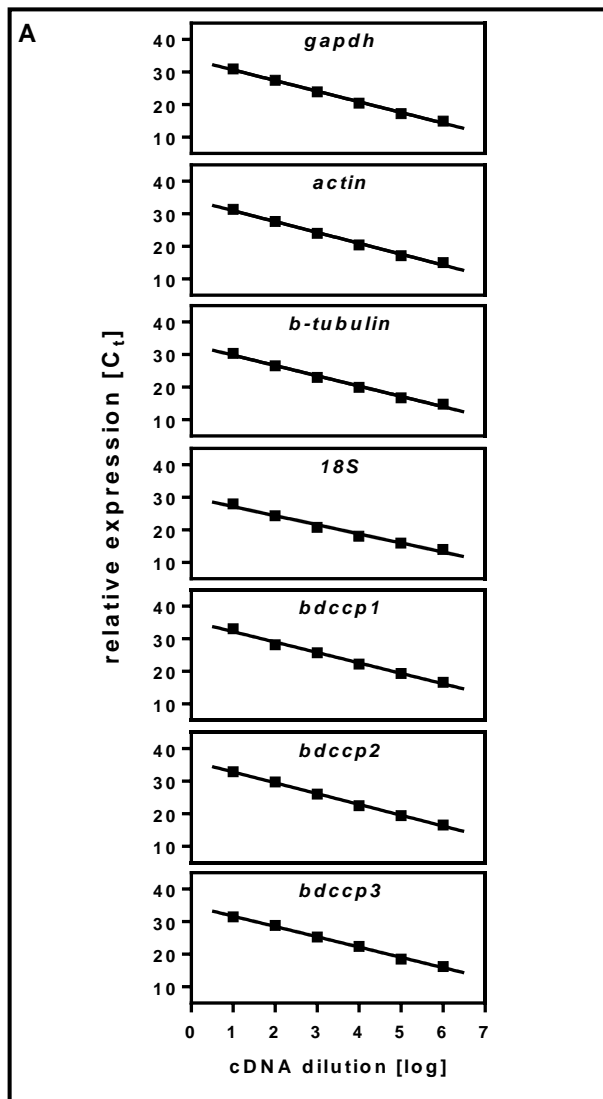

**B**

| gene             | R2    | slope  | efficiency [%] |
|------------------|-------|--------|----------------|
| <i>gapdh</i>     | 0.994 | -3.182 | 106.171        |
| <i>actin</i>     | 0.994 | -3.261 | 102.616        |
| <i>b-tubulin</i> | 0.991 | -3.161 | 107.203        |
| 18S              | 0.994 | -3.182 | 106.214        |
| <i>bdccp1</i>    | 0.990 | -3.199 | 105.413        |
| <i>bdccp2</i>    | 0.998 | -3.318 | 100.147        |
| <i>bdccp3</i>    | 0.997 | -3.160 | 107.227        |

Supplement: Additional file 3: Figure S2. — Optimization of qRT-PCR. Standard curves of reference and target genes (A) and qRT-PCR parameters (B). Ct = cycle threshold, R2 = correlation coefficient. (PDF 181 kb) [file 13071_2016_1731_MOESM3_ESM.pdf]

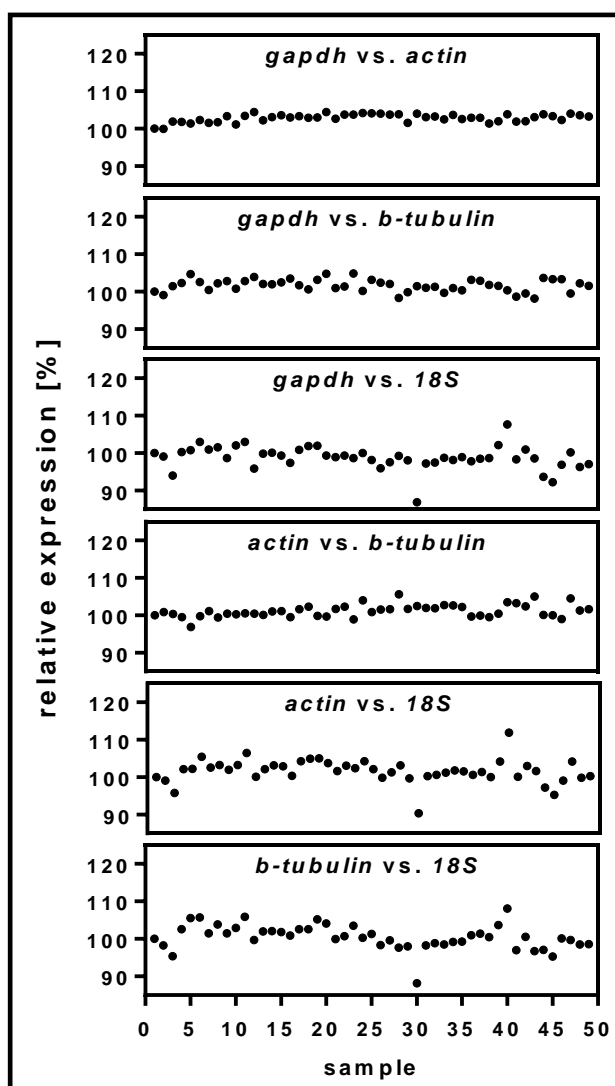

Supplement: Additional file 4: Figure S3. — Comparison of stability of reference genes. Reference genes were evaluated by comparisons of all reference genes using Ct values. The first sample in each gene analysis was set at 100 % and all other values were normalized to this. (PDF 66 kb) [file 13071_2016_1731_MOESM4_ESM.pdf]

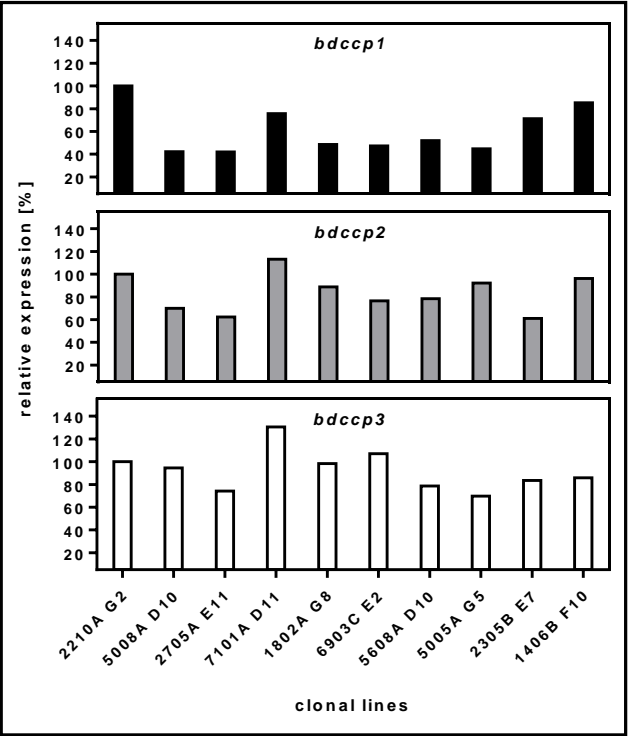

Supplement: Additional file 5: Figure S4. — Relative expression of bdccp genes in various bovine clonal lines of B. divergens. Gene expression was normalized using the gapdh reference gene. Expression in the clone 2210A G2 was set at 100 % and all other values were expressed relative to this. (PDF 49 kb) [file 13071_2016_1731_MOESM5_ESM.pdf]

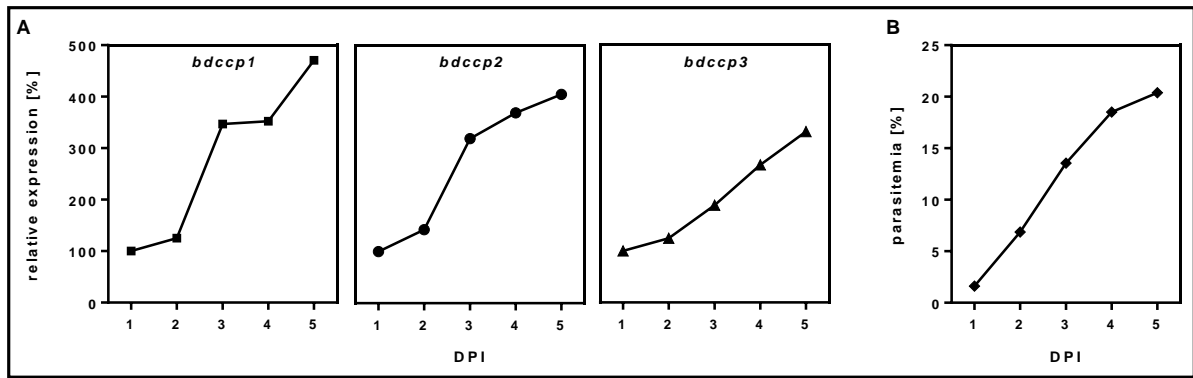

Supplement: Additional file 6: Figure S5. — Continuous culture growth. Relative expression of bdccp genes (A) and parasitemia levels (B) during the continuous growth of B. divergens clone 2210A G2. Gene expression was normalized using the gapdh reference gene. The expression in the highest individual replicate 1 DPI was set at 100 % and all other values were expressed relative to this. (PDF 48 kb) [file 13071_2016_1731_MOESM6_ESM.pdf]
